# Landlord Behavior After Receiving Pediatrician-Generated Letters to Address Poor Housing Conditions

**DOI:** 10.1001/jamanetworkopen.2021.28527

**Published:** 2021-10-06

**Authors:** Yonit Lax, Gabriel Cohen, Amy Mandavia, Steven Morrin, Jeffrey R. Avner

**Affiliations:** 1 Department of Pediatrics, Maimonides Children’s Hospital, Brooklyn, New York; 2 Department of Pediatrics, College of Medicine, SUNY Downstate Health Sciences University, Brooklyn, New York; 3 Department of Population Health, Maimonides Medical Center, Brooklyn, New York; 4 College of Medicine, SUNY Downstate Health Sciences University, Brooklyn, New York

## Abstract

This cohort study examines whether providing a pediatrician-generated letter regarding patients’ poor housing conditions can encourage landlords to improve those conditions.

## Introduction

Indoor environmental exposures are detrimental to health outcomes and contribute to childhood morbidity; however, addressing these exposures remains a challenge in pediatric care.^1,2^ To mitigate morbidity associated with poor housing conditions, interventions using community health workers,^3^ mobility experiments relocating families,^4^ and medical-legal partnerships have proven successful.^5^ However, these interventions are often constrained by availability of funding, resources, and time.^1^ Using physician-generated letters is 1 strategy applied in medical-legal partnerships that does not require additional funding or resources.^6^ In this study, we examined the association between physician-generated housing letters and improvement of poor housing conditions.

## Methods

In this cohort study conducted from April through December 2019, an electronic health record–based social determinants of health screening tool and referral model was implemented in 3 urban hospital-based pediatric primary care sites (Pediatrics at 57th Street, Pediatrics at 7th Avenue, and Newkirk Family Health Center in Brooklyn, New York). Housing, food, and child care or developmental concerns were among the social determinants of health assessed by the screening tool. Families were defined as having poor housing conditions if they gave an affirmative answer to the question “Are you currently having any housing problems (overcrowding, roaches, rodents, utilities, mold, lead) that your landlord is not helping you with?” Patients with poor housing conditions were offered a physician-generated letter (available through the electronic health record) advocating for the landlord to fix the conditions and explaining the health consequences they might cause. Maimonides Medical Center Institutional Review Board deemed this study exempt from review and waived the requirement for informed consent because it was conducted as a quality improvement measure as part of health care operations. This study followed the Strengthening the Reporting of Observational Studies in Epidemiology (STROBE) reporting guideline.

All patients at the pediatric primary care clinics who were screened for social determinants of health were eligible to participate in this study. Data on self-reported primary language were collected from the electronic health record rather than data on race and ethnicity because language affected the administration of the initial quality improvement surveys. Caregivers of patients who reported poor housing conditions and received a physician-generated letter between April and October 2019 were surveyed about the landlords’ actions 2 to 6 months later.

The outcomes of interest were (1) rates of poor housing conditions and (2) resolution of the poor conditions. χ^2^ tests were performed, with 2-sided *P* < .01 deemed to be significant, and mean difference with 95% CIs were calculated. SPSS software, version 27 (IBM Corporation) was used to analyze the data.

## Results

Between April and October 2019, 233 of 2480 (9%) families who were screened for social determinants of health needs had poor housing conditions. Of these families, 127 (55%) requested and received a physician-generated letter advocating for housing repairs. Demographic characteristics of participating families are reported in Table 1.

**Table 1.** Demographic Characteristics of Participating Families

Characteristic	No. (%)			
	Family accepted housing letter	Completed phone survey	Gave letter to landlord	Landlord acted to resolve issue
Total No. of families	127 (100)	96 (76)	35 (36)	31 (89)
Patient age, y				
0-5	70 (55)	54 (56)	22 (63)	19 (61)
6-11	30 (24)	20 (21)	6 (17)	5 (16)
11-18	27 (21)	22 (23)	7 (20)	7 (23)
Language^a^				
Spanish	57 (45)	46 (48)	19 (54)	17 (55)
English	59 (46)	43 (45)	15 (43)	13 (42)
Bengali	7 (6)	4 (4)	0	0
Cantonese	2 (2)	2 (2)	1 (3)	1 (3)
American Sign Language	1 (0.5)	1 (1)	0	0
Albanian	1 (0.5)	0	0	0

^a^ Primary language was self-reported and collected from the electronic health record.

A total of 96 (76%) families completed the follow-up telephone survey between June and December 2019. Of the 35 families (36%) who reported giving the letter to their landlords, 31 (89%) reported that the landlord acted to resolve the issue, and 26 (74%) reported complete resolution of the concern. The families who received the housing letter were significantly more likely than families who did not receive housing letters to experience food insecurity (31.5% vs 17.3%; *P* < .001), child care or developmental concerns (26.8% vs 12.1%; *P* < .001), and problems with finances or benefits (26.0% vs 9.8%; *P* < .001) (Table 2). For the 149 families who had a subsequent follow-up visit, there was no significant difference in those without poor housing conditions between families who did vs did not receive letters (44 of 61 [72%] vs 63 of 88 [72%]).

**Table 2.** Social Determinants of Health Needs of Families Who Received Housing Letters

Need	Housing letter requested, No. (%)		Mean difference, % (95% CI)	*P* value
	Yes (n = 127)	No (n = 2376)		
Food insecurity	40 (31.5)	410 (17.3)	14.2 (7.4 to 21.1)	<.001
Adult education	74 (58.3)	1198 (50.4)	7.8 (−1.1 to 16.8)	.04
Child care or developmental concern	34 (26.8)	288 (12.1)	14.7 (8.7 to 20.6)	<.001
Financial or benefit need^a^	33 (26.0)	233 (9.8)	16.2 (10.7 to 21.7)	<.001

^a^ Benefits included Special Supplemental Nutrition Program for Women, Infants, and Children; Supplemental Nutrition Assistance Program; medical insurance; daycare vouchers; and Supplemental Security Income.

## Discussion

Integrating the letter template into the electronic health record allows for a quick intervention, providing families with a simple way to attempt to remediate their housing conditions before resorting to more time-intensive solutions, such as moving or legal action.^6^ Furthermore, most families who gave letters to their landlords reported that the process led to landlord action and improved housing conditions.

Limitations of this study include selection bias, response bias, and being a single-center study with a small sample size. In addition, this study was not designed to assess whether families who did not receive the letter had resolution of their conditions. Nevertheless, our findings suggest that physician-generated housing letters have the potential to be an effective intervention to address poor housing conditions.
